# Pre-sleep treatment with galantamine stimulates lucid dreaming: A double-blind, placebo-controlled, crossover study

**DOI:** 10.1371/journal.pone.0201246

**Published:** 2018-08-08

**Authors:** Stephen LaBerge, Kristen LaMarca, Benjamin Baird

**Affiliations:** 1 Lucidity Institute, Pahoa, HI, United States of America; 2 Wisconsin Institute for Sleep and Consciousness, Department of Psychiatry, University of Wisconsin–Madison, Madison, WI, United States of America; Associazione OASI Maria SS, ITALY

## Abstract

Lucid dreaming is a remarkable state of consciousness in which one is aware of the fact that one is dreaming while continuing to dream. Based on the strong relationship between physiological activation during rapid eye-movement sleep and lucid dreaming, our pilot research investigated whether enhancing cortical activation via acetylcholinesterease inhibition (AChEI) would increase the frequency of lucid dreams and found AChEI to be a promising method for lucid dream induction. In the current study we sought to quantify the size and reliability of the effect of AChEI on lucid dreaming, dream recall and dream content as well as to test the effectiveness of an integrated lucid dream induction protocol which combined cholinergic stimulation with other methods for lucid dream induction. Participants (N = 121) with high dream recall and an interest in lucid dreaming were randomly assigned counterbalanced orders of 3 doses of galantamine (0, 4 and 8 mg). On 3 consecutive nights, they awoke approximately 4.5 hours after lights out, recalled a dream, ingested the capsules and stayed out of bed for at least 30 minutes. Participants then returned to bed and practiced the Mnemonic Induction of Lucid Dreams technique while returning to sleep. The percentage of participants who reported a lucid dream was significantly increased for both 4 mg (27%, odds ratio = 2.29) and 8 mg doses (42%, odds ratio = 4.46) compared to the active placebo procedure (14%). Galantamine also significantly increased dream recall, sensory vividness and complexity (*p*<0.05). Dream recall, cognitive clarity, control, positive emotion, vividness and self-reflection were increased during lucid compared to non-lucid dreams (*p*<0.0001). These results show that galantamine increases the frequency of lucid dreams in a dose-related manner. Furthermore, the integrated method of taking galantamine in the last third of the night with at least 30 minutes of sleep interruption and with an appropriately focused mental set is one of the most effective methods for inducing lucid dreams available today.

## Introduction

During a lucid dream one becomes aware that one is dreaming while continuing to dream. In this remarkable state of consciousness, one can reflect rationally and engage in purposeful, volitional actions within the dream. For example, lucid dreamers can recognize that their current sensory experience is a mental construction unrestricted by the usual limits of the waking physical world and often recall details about waking conditions. These details can include autobiographical memories from waking, as well as preset intentions to carry out specific actions in the dream [1, 2]. Awareness of dreaming, and the increased volition and autobiographical memory that often accompany it, occurs while the dreamer remains in unequivocal REM sleep [3], immersed in a dream environment that can appear strikingly realistic. Lucid dreaming presents a unique opportunity for entertainment, exploration and personal growth [4]. Furthermore, the scientific study of lucid dreaming opens novel experimental approaches for studying the psychophysiology of REM sleep that have the potential to expand our understanding of the relationship between consciousness and brain activity [5]. However, while the majority of people report having experienced a lucid dream at least once in their lives, for most people lucid dreams occur very infrequently [6]. Accordingly, the development of reliable methods of inducing lucid dreams is an important focus of research [4, 7–13].

In his dissertation, LaBerge [14] developed one of the first reliable techniques for lucid dream induction based on scientific research, referred to as the Mnemonic Induction of Lucid Dreams (MILD). The purpose of MILD is to increase the frequency of lucid dreams using the intention to remember to do something in the future, a behavior now termed prospective memory. To effectively practice MILD requires good dream recall. One begins by remembering a dream, either from a previous night or, ideally, from which one has just awakened. One then identifies an anomaly within the dream that is characteristic of one’s dreams (called a “dreamsign” [1]). Then, one visualizes returning to the dream, and rehearses recognizing that this anomaly only occurs in dreams, thereby becoming lucid. During this visualization, the practitioner mentally recites, “The next time I’m dreaming I want to remember to recognize that I’m dreaming.” In addition, the technique involves visualizing carrying out a preselected action when one becomes lucid. A meditative focus is required to use MILD effectively. The practitioner repeatedly lets go of distracting thoughts, returning focus on the visualization of recognizing the dreamsign, becoming lucid, and performing the preselected goal, consistently until falling back to sleep. In a case report, LaBerge [7] found that MILD was approximately 800% more effective than autosuggestion for inducing signal-verified lucid dreams in the sleep laboratory. Follow-up studies have confirmed that MILD can increase lucid dream frequency [15].

Another lucid dream induction technique is to use a sensory stimulus as a cue, such as a flashing light applied over the sleeper’s eyes via a sleep mask during REM sleep [8, 10]. Such stimuli are frequently incorporated into an ongoing dream, and with proper mental preparation, individuals can learn to identify the stimulus as a cue and thereby become lucid. Again, prospective memory—the intention to recognize the cue—is required for success with this technique of lucid dream induction. A third approach to lucid dream induction arose from reports that lucid dreaming was more likely after engaging in certain activities during the night, such as meditation, sexual intercourse or reading (e.g., [16, 17]). Given the diversity of these activities, LaBerge [7] suggested that it was not the particular activity but rather the period of alert wakefulness that made lucid dreaming more likely during subsequent sleep. A period of 10–15 minutes of wakefulness was therefore incorporated into the MILD technique [7]. Subsequent studies showed that combining MILD with a longer period of wakefulness of either 30 to 60 minutes late in the sleep cycle was associated with even higher increases in lucid dream frequency in the ensuing sleep period [18]. Consistent with these findings, a recent study found that participants who practiced a combination of MILD and sleep interruption nightly for two weeks demonstrated increased lucid dreaming frequency [19].

In earlier research we found that lucid dreams tend to occur during periods of increased physiological activation during REM sleep, and that measures of phasic central nervous system activation, such as increased eye movement density, are associated with lucid dreams [5, 20, 21]. Therefore, increasing physiological activation in REM sleep is another potentially fruitful avenue to explore for stimulating lucidity in dreams. Given the strong evidence that REM sleep is modulated by acetylcholine [22–24] and furthermore that acetylcholinesterease inhibition increases REM sleep phasic activity [25], we tested whether enhancing cortical activation via cholinergic stimulation would increase the frequency of lucid dreaming. In an initial pilot study [26], we evaluated the influence of either 0 mg (placebo), 5 mg, or 10 mg of donepezil (Aricept®), an acetylcholinesterease inhibitor (AChEI), administered at bedtime in a within-subjects counter-balanced order on three nights in a small group of individuals with prior experience in lucid dreaming. Nine of the ten participants (90%) reported one or more lucid dreams on the experimental nights on donepezil, with only one participant reporting a lucid dream on a placebo night (*p*<0.01).

In the current study we sought to quantify the size and reliability of the effect of AChEIs on lucid dreaming, dream recall and dream content. We evaluated the effect of galantamine, a fast acting AChEI with a mild side effect profile, in a large group of individuals (N = 121) with an interest in lucid dreaming. We tested the effect of galantamine taken during a period of sleep interruption after approximately the third REM cycle. This design allowed us to examine the combined effectiveness of an integrated lucid dream induction protocol, incorporating sleep interruption and the MILD technique together with cholinergic enhancement. Data was collected using a double blind, placebo-controlled, cross-over design across six lucid dreaming training programs.

## Materials and methods

### Participants

Volunteers were recruited from a high-interest group attending one of six, 8-day training programs on lucid dreaming, titled “Dreaming and Awakening”, presented by the Lucidity Institute. The study was approved by the Lucidity Institute research ethics committee and was conducted in accordance with the Declaration of Helsinki. Participation in the study was voluntary. All participants provided signed informed consent prior to the experiment. Participants’ responses were treated confidentially and anonymously. Exclusion criteria included asthma, taking beta-blockers, severe mental illness or cardiac arrhythmias. However, no exclusions were made for biomedical reasons as no participants presented with any of the exclusionary criteria. The details, study rationale, and possible side effects of cholinergic stimulation, as described above, were explained, including the possibility of unpleasant side effects such as insomnia or nausea. Participants were aware that on at least one of the experiment nights they would be receiving an active dose of an over-the-counter supplement. All workshop participants who met the eligibility criteria and wished to participate in the experiment were included in the study. Extensive piloting of the procedures in previous workshops allowed us to hold all aspects of the protocol constant throughout the entire duration of the study.

One hundred twenty nine participants participated in the study. Eight participants were not included in analysis due to not completing at least two nights of the experiment or following instructions, including one participant who discontinued the study due to nausea, and one to avoid the sleep disturbance from completing the nightly procedures. The final group included in the analysis consisted of 121 participants (63 males, 58 females), age 43 ± 12, 19–75 years [mean ± SD, range]. Participants reported median rates of 1 dream recalled per night and 3–5 lucid dreams recalled per year, similar to pre-experiment estimates of the number of lucid dreams recalled in the last six months (median = 2), and most recalled in any 6-month period (median = 3). Ten participants reported no previous lucid dreams.

### Procedures

Participants engaged in an integrated lucid dream induction protocol, which combined sleep interruption and the Mnemonic Induction of Lucid Dreams (MILD) technique together with cholinergic enhancement. As part of the workshop, participants attended lectures about lucid dreaming, which explained what lucid dreams are, reviewed examples of lucid dreams, and provided time for discussion and questions. Participants therefore obtained a thorough conceptual understanding of lucid dreaming during the training workshop before the experiment began. As part of their training in MILD (described above), they learned specific strategies for recognizing cues that they are dreaming [27], prolonging the lucid dream state [28], and responding adaptively to nightmares and sleep paralysis [4].

**Fig 1:**
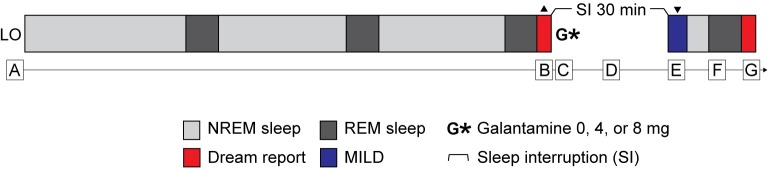
Schematic diagram of experimental procedure. (Note: figure not to scale, timing approximate). **A.** LO: Lights Out. Sleep for approximately 3 REM cycles. **B.** DREC: Recall and memorize dream upon awakening to use with MILD procedure at **E**. **C.** Ingest galantamine capsules. (All participants received all three doses (0, 4, and 8 mg) in one of 6 counterbalanced orders.) **D.** Sleep interruption (out of bed for 30 min). Engage in quiet activity with focus on lucid dreaming. (“Wake back to bed") **E.** Return to sleep practicing MILD using the dream recalled at **B. F.** Experimental Nap(s). **G.** REPORT: On awakening, dream recall, content scales, and full reports for lucid dreams.

Participants were instructed on the nightly reporting procedures and the protocol for sleep interruption, which included a modified sleep schedule in which participants interrupted their sleep with a 30-minute period of wakefulness after approximately 4.5 hours of sleep (a rough estimate of 3 REM periods) [see Fig 1 for a schematic diagram of the experimental procedure]. The 30-minute sleep interruption period was selected based on previous research, as noted above, showing that 30 minutes of sleep interruption is more effective than shorter amounts of time [18]. We did not set the sleep interruption period for longer than 30 minutes because it does not lead to further increases in lucid dream frequency. Furthermore, galantamine reaches peak serum concentration (Cmax) approximately 1 hour after oral ingestion [29], and we wanted to align Cmax to occur during the next sleep (and dream) period after allowing participants time to fall back to sleep. Participants practiced MILD while returning to sleep for all awakenings after the sleep interruption period. 94 participants also wore a sleep mask which recorded physiological variables and provided additional memory cues during sleep [8, 10] (see S2 Appendix). Participants who opted to sleep with the mask were required to use it on all three nights of the experiment after the sleep interruption period. Participants used a paper-pencil form to track their nightly experiences and time variables, including their lights out time, the time they started the sleep interruption period, the length of the sleep interruption period, the time they ingested the capsules, and their rising time in the morning. Participants were given two nights prior to the experiment to practice the induction and reporting protocol. The experiment was initiated on the fifth night of the workshop.

Participants were randomly assigned subject numbers. On each night (A, B, C), they received a coded packet, according to their subject numbers and appropriate night, containing capsules with 0 mg (G0), 4 mg (G4), or 8 mg (G8) of galantamine hydrobromide (Life Enhancement Products Galantamind; S2 Appendix). Numbered packets were arranged to provide a counterbalanced order of doses; however, the participants and experimenters were unaware which dose individuals would be receiving on a particular night (double-blind design). Each night, participants awoke after approximately 4.5 hours of sleep, recalled and memorized a dream, ingested the capsules, and then remained awake for the sleep interruption period while engaged in a quiet wakeful activity of their choice (e.g., reading about lucid dreaming or recording or reviewing their dreams). Participants then returned to bed, practicing MILD until they fell asleep. For all awakenings after the period of sleep interruption, participants completed the report form and recorded the time they woke up and the type of dream.

As noted above, lucidity can arise during an ongoing dream (often through the recognition of a cue or an anomaly indicating that one is dreaming) or, less commonly, by maintaining awareness while transitioning directly from the waking state into a dream. These two types of lucid dream onset are referred to respectively as dream-initiated lucid dreams (DILDs) and wake-initiated lucid dreams (WILDs) [15, 20]. On the dream report forms, participants categorized their dream as none (did not recall a dream), non-lucid dream, DILD or WILD. If participants recalled a dream, they rated their extent of dream recall and rated the dream on several dimensions of conscious experience, including sensory vividness, clarity of thinking, negative and positive emotion, bizarreness, complexity, self-reflection, public self-consciousness, and degree of dream control (all on a 0 to 4 scale with 0 = none, 4 = high). Participants also reported "odd somatic sensations", such as strange bodily feelings, paralysis, tingling, vibrations, etc. as well as any perceived side effects (S2 Appendix). For lucid dreams participants wrote a full narrative report of the dream. In the reports, participants were asked to specify the point in the dream report at which they became lucid as well as how they knew they were dreaming (e.g., if it was triggered by a particular event or associated with a specific thought during the dream). Participants also gave a follow-up oral report of all lucid dreams the following morning. Two complementary criteria had to be met in order for a dream report to be classified as lucid. First, the participant himself or herself had to rate their dream as lucid (either a DILD or a WILD). Second, dream reports had to include a reference to the current state as a dream (for example, “It was at this point I knew it was a dream”; “I realized I am dreaming”; “I said aloud, ‘*This is a dream*.’”).

### Statistical analysis

Linear mixed models were used to account for repeated measures with varied numbers of repeated observations within subjects. The model for evaluating the effect of DOSE (*active placebo (G0)*, *galantamine 4 mg (G4)*, *galantamine 8 mg (G8))* on STATE (*lucid*, *non-lucid*) used restricted maximum likelihood estimation (REML) and included DOSE and NIGHT (*1*, *2*, *3*) as fixed effects and STATE as the outcome variable. The model for responses to the dimensions of consciousness (DIMs) scale used restricted maximum likelihood estimation (REML) and included participant as a random factor, and NIGHT, DOSE, and STATE as fixed effects. As questionnaire responses as well as outcome measures were not normally distributed, we used nonparametric bootstrapping for all significance tests. (We also evaluated the effect of DOSE on STATE using binary logistic regression, for which identical significance thresholds were obtained compared to the bootstrap method). Hypothesis testing of regression coefficients (pairwise tests) from the mixed models was obtained by the following steps: *i*) constructing a model based on the null hypothesis of no differences between STATE (*H*_*0*_), *ii*) resampling with replacement the distribution of the response residuals under the null model, reconstructing a bootstrap *y* response vector, and refitting the *H*_*1*_ model to the bootstrap response vectors to generate 10,000 bootstrap estimates of the regression coefficients (*ß*) under *H*_*0*_, and *iii*) comparing the observed value of *ß* against the null bootstrap distribution (two-tailed frequentist *p*-value). Mixed model construction and mixed model bootstrapping were performed with the lme4 package [30] in the R environment (R Development Core Team, 2006). Mixed model fixed effects were assessed by means of a bootstrap likelihood ratio test on mixed effects models (PBmodcomp in R) specified with maximum likelihood estimation (MLE). We tested 10 DIMs variables for both STATE and DOSE effects (40 total tests); we therefore corrected the type I error rate for these multiple comparisons with the Bonferroni correction (critical α = 0.05/40 = 0.0012).

### Bayesian classification

We evaluated the extent to which STATE (*lucid*, *non-lucid*) could be classified using a Bayesian classification procedure developed and validated by Allen et al. [31]. We performed a 2-step split-half classification: in the first half, we used binary logistic regression to determine the content variables with the highest predictive value (regression coefficients); in the second half, we then used Bayesian combination of these multiple indicators (predictor variables) to calculate the Bayesian Posterior Probability (BPP) (i.e., the probability that a dream was either lucid or non-lucid given an observed combination of indicators, for example high control and high positive emotionality). The cutpoint for each variable was set at 2 on the 0–4 scale; thus, scores of 0–2 were categorized as low and scores of 3–4 were categorized as high. The BPP is equal to the proportion of dream reports associated with lucid dreams that show the combination of indicators divided by the proportion of all trials (lucid and non-lucid) showing the combination of indicators (1.00 = perfect classification).

## Results

On average, participants reported a total sleep period (lights out to rising time) of 8.1 ± 1.3 [mean ± SD] hours. The sleep interruption period was started on average at 4.54 ± 0.86 [mean ± SD] hours after lights out and lasted 36 ± 18 [mean ± SD] minutes. Lights out to rising time after the sleep interruption period lasted an average of 3.3 ± 1.1 [mean ± SD] hours. In total, 485 dreams were reported: 85 (17%) were DILDs, 32 (7%) were WILDs, and 368 (76%) non-lucid dreams.

### Lucidity

**Fig 2:**
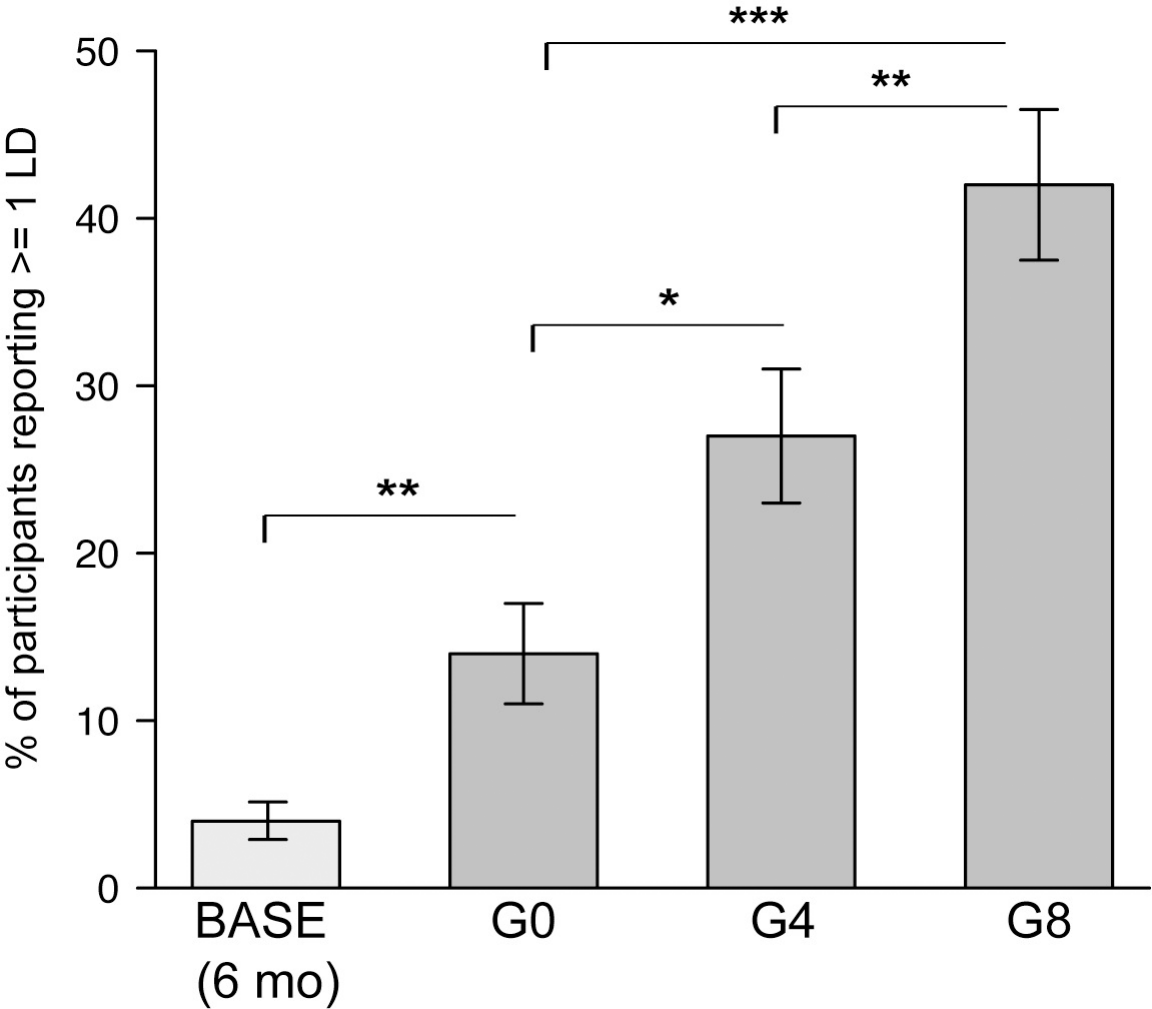
Percent of participants (N = 121) reporting at least one lucid dream (LD) on returning to sleep following ingestion of one of three masked doses of galantamine (0 mg [G0], 4 mg [G4], and 8 mg [G8]) prior to 30–40 minutes out of bed. The baseline estimate (“BASE”, 4%) of lucid dreaming frequency for one night was calculated from the self-reported estimates of how many LDs participants experienced in the previous six months, divided by 180. [95% CI computed by resampling.] Error bars show estimated standard error of the conditional means. Asterisks indicate statistically significant differences between conditions: * p<0.05; ** p<0.01; *** p<0.001.

75 participants (62%) reported one or more lucid dreams during the study—69 (57%) on one or more nights with galantamine and 17 (14%) with the active placebo procedure (including sleep interruption and MILD); 33 (27%) participants with the 4 mg dose, and 51 (42%) participants with the 8 mg dose [Fig 1; Table 1; see S1 Appendix for examples of lucid dreams participants reported during the study]. There was a main effect of galantamine on lucidity [likelihood ratio: 25.54, *p*<0.001]. Significantly increased incidence of lucid dreaming was observed for both 4 mg (*ß* = 0.12, *p* = 0.03, odds ratio = 2.29, 95% CI = 1.19–4.38) and 8 mg doses (*ß* = 0.28, *p*<0.0001, odds ratio = 4.46, 95% CI = 2.38–8.35) compared to the active placebo [Fig 2]. The 8 mg dose resulted in significantly higher incidence of lucid dreams compared to the 4 mg dose (*ß* = 0.16, *p* = 0.004, odds ratio = 1.94, 95% CI = 1.13–3.33). A dose response analysis confirmed a dose-related response (linear trend) for lucid dreaming probability by galantamine exposure (Mantel-Haenszel *χ*^2^ for linear trend = 23.03, *p* = 0.000001). Compared to the expected frequency of lucid dreams (4%, based on self-reported frequency of lucid dreaming during the 6 months prior to the experiment), lucid dream frequency was approximately three times higher (14%, *p* = 0.01) for the active placebo procedure (including MILD and sleep interruption). For the combined protocol with 4 mg galantamine, lucid dream frequency was approximately 6 times higher than expected (27%, *p*<10^−5^), and approximately 9 times higher than expected for the combined protocol with 8 mg galantamine (42%, *p*<10^−10^) [Table 1].

**Table 1:** Percent of 121 participants reporting at least one lucid dream in a double-blind, randomized crossover design comparing pre-sleep administration of three doses of Galantamine (0 mg, 4 mg, and 8 mg).

	Expected Prior6mo	0 mg	Observed 4 mg	8 mg
**% (N = 121)**	**4.3%**^†^	**14.1%**	**27.3%**	**42.2%**
	[3–6]	[8–21]	[20–36	[[33–52]
**% increase**	**-**	**227%**	**535%**	**881%**
		[86–388]	[365–737]	[667–1110]

^†^*Note*: expected percentages based on self-reported frequency of lucid dreaming during the 6 months prior to the experiment. Brackets indicate 95% CI. Significant differences were observed between all 4 conditions (*p* < 0.05).

There was no main effect of night on lucidity [likelihood ratio: 1.33, *p* = 0.52], indicating that participants were not more likely to have a lucid dream on subsequent nights of the experiment independently of other effects. Furthermore, there was no effect of dose order on the likelihood of having a lucid dream (*F*(1,5) = 0.31, *p* = 0.90), supporting that consecutive nights were not too close together. Across all dream reports, participants reported a total of 15 DILDs on placebo, 27 DILDs on the 4 mg dose and 43 DILDs on the 8 mg dose, while 3 WILDs were reported on placebo, 10 WILDs on the 4 mg dose and 19 WILDs on the 8 mg dose. Both DILDs (*ß* = 0.096, *p* = 0.006) and WILDs (*ß* = 0.063, *p* = 0.004) were significantly increased on an active dose of galantamine compared to placebo.

### Age and gender effects

Overall there was no significant association between age and the frequency of lucid dreams reported during the study (*ß* = -0.003, *p* = 0.09). Additionally, there was no difference in age between participants who had a lucid dream on an active dose of galantamine and those who did not (*t*(119) = 1.16, *p* = 0.25). Older individuals did, however, report overall reduced levels of dream recall (*ß* = -0.016, *p* = 0.02). There were no gender differences in lucid dream frequency during the study overall (males = 36, females = 39, *t*(119) = 1.14, *p* = 0.26) or on an active dose of galantamine (females: 33 out of 58 participants (56.8%); males: 36 out of 63 participants (57.1%)).

### Prior experience with lucid dreaming

Participants with higher baseline levels of lucid dreams, as estimated by the number of lucid dreams in the last 6 months, were more likely to have lucid dreams during the experiment overall (*ß* = 0.04, *p* = 0.0001). Furthermore, individuals who had a lucid dream on an active dose of galantamine reported higher baseline lucid dreaming frequency than those who did not have a lucid dream on the active dose [*t*(119) = 2.65, *p* = 0.009]. As noted above, 10 participants reported never experiencing a lucid dream before the experiment. Four of these participants (40%) were able to achieve lucidity during the study, all with the 8 mg dose of galantamine. This was similar to the 42% success rate (47/111) with the same dose for individuals reporting at least one lucid dream prior to the study. No difference in the success rate was observed between individuals who reported a lucid dream prior to the study and those who had never had a lucid dream (*χ*^2^ (1,N = 121) = 1.28, *p* = 0.25).

### MILD and sleep interruption

Participants who practiced MILD for at least 10 minutes (the recommended minimum amount of time) were more likely to have a lucid dream (*ß* = 0.09, *p*<0.01, one-tailed). However, the total number of minutes practicing MILD did not predict success in having a lucid dream (*ß* = 0.003, *p* = 0.80). Participants were more likely to have a lucid dream if they engaged in at least 30 minutes of sleep interruption (*ß* = 0.11, *p* = 0.03, one-tailed; S2 Appendix), confirming the value of sleep interruption for lucid dreaming induction.

### Dream cognition and recall

**Fig 3:**
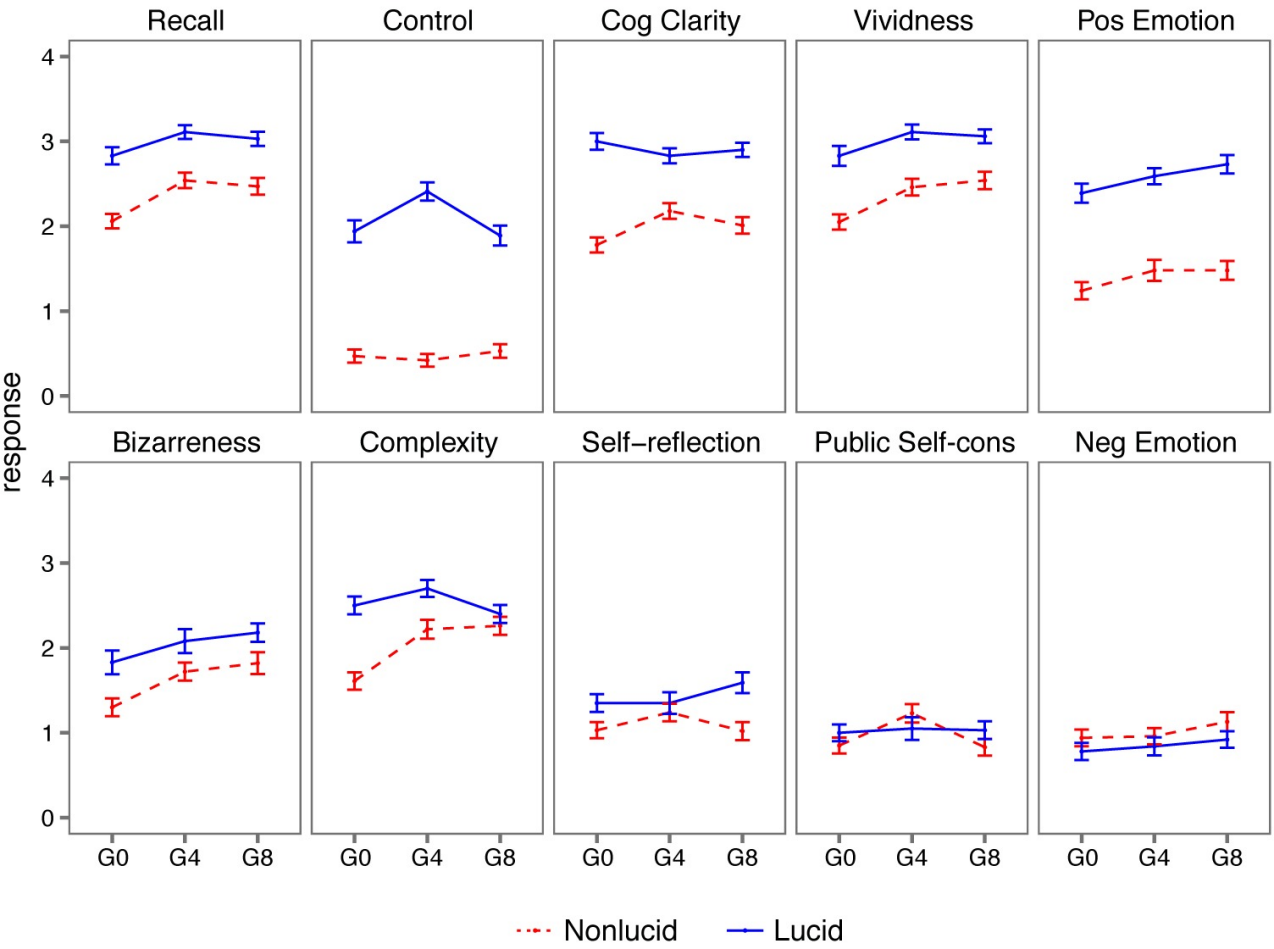
Responses to dimensions of consciousness (DIMs) questionnaire separated by lucid and non-lucid dreams and galantamine dose (G0 = 0 mg; G4 = 4 mg; G8 = 8 mg). Points and error bars show the mean and standard error of the mean.

During lucid compared to non-lucid dreams, participants reported significantly higher sensory vividness, clarity, positive emotion, control, and self-reflection (all *p*<0.05, Bonferroni corrected; Table 2, Fig 3). Increased complexity and bizarreness and decreased negative emotion were also observed (*p*<0.05), though these differences did not survive correction for multiple comparisons. Compared to placebo, both 4 mg and 8 mg doses of galantamine significantly increased sensory vividness and environmental complexity (all *p*<0.05, Bonferroni corrected; Table 3, Fig 3). Increased bizarreness was observed under the 8 mg dose compared to placebo (*p*<0.05, Bonferroni corrected); 4 mg and 8 mg doses did not significantly differ on any dimension (all *p*>0.05; Table 3). Dream recall was higher for lucid compared to non-lucid dreams (*ß* = 0.57, *p*<0.0001), and was higher under both the 4 mg (*ß* = 0.43, *p*<0.0001) and 8 mg (*ß* = 0.39, *p*<0.0001) doses compared to placebo. Lucid dreams on an active dose of galantamine did not differ from lucid dreams on the placebo dose for any content dimension (all *p*>0.05).

**Table 2:** Dimensions of consciousness (DIM) for lucid and non-lucid dreams.

DIM	NLD [M (SD)]	LD [M (SD)]	*Beta*	*p-value*
Recall	2.36 (1.01)	3.03 (0.92)	0.57	<0.0001*
Vividness	2.36 (1.08)	3.04 (0.98)	0.61	<0.0001*
Clarity	2.00 (1.03)	2.89 (0.96)	0.85	<0.0001*
Neg Emotion	1.01 (1.13)	0.87 (1.10)	-0.26	0.02
Pos Emotion	1.41 (1.24)	2.63 (1.15)	1.12	<0.0001*
Bizarreness	1.62 (1.27)	2.09 (1.37)	0.31	0.01
Complexity	2.04 (1.21)	2.51 (1.14)	0.34	0.002
Control	0.47 (0.84)	2.06 (1.29)	1.61	<0.0001*
Self-reflection	1.10 (1.12)	1.48 (1.33)	0.48	<0.0001*
PubCons	0.98 (1.12)	1.03 (1.24)	0.06	0.55

Note

* denotes significant differences between conditions after correcting for multiple comparisons (Bonferroni correction). NLD = non-lucid dream, LD = lucid dream.

**Table 3:** Dimensions of consciousness (DIM) by galantamine dose (0 mg (G0), 4 mg (G4) and 8 mg (G8)).

	Mean (SD)			G4 > G0				G8 > G0			G4 > G8	
DIM	PL	G4	G8	*Beta*		*p*-value		*Beta*	*p*-value		*Beta*	*p*-value
Lucidity	0.14 (0.36)	0.27 (0.44)	0.42 (0.49)	0.12		0.03		0.28	<0.0001*		0.16	0.004
Recall	1.56 (1.26)	2.10 (1.38)	2.25 (1.35)	0.43		<0.0001*		0.39	<0.0001*		0.03	0.64
Vividness	2.16 (1.05)	2.60 (1.09)	2.72 (1.08)	0.35		0.0002*		0.41	<0.0001*		-0.06	0.46
Clarity	1.94 (1.07)	2.33 (1.05)	2.30 (1.10)	0.31		0.002		0.19	0.04		0.12	0.15
Neg Emotion	0.92 (1.08)	0.93 (1.07)	1.06 (1.19)	0.06		0.55		0.21	0.08		-0.13	0.22
Pos Emotion	1.40 (1.19)	1.73 (1.36)	1.91 (1.35)	0.19		0.09		0.26	0.02		-0.06	0.54
Bizarreness	1.37 (1.22)	1.80 (1.26)	1.94 (1.36)	0.33		0.009		0.50	<0.0001*		-0.16	0.18
Complexity	1.73 (1.16)	2.32 (1.21)	2.31 (1.17)	0.61		<0.0001*		0.56	<0.0001*		0.04	0.65
Control	0.67 (1.07)	0.86 (1.23)	0.99 (1.22)	0.06		0.44		0.002	0.97		0.06	0.44
Self-reflection	1.07 (1.06)	1.27 (1.20)	1.22 (1.26)	0.21		0.03		0.08	0.40		0.13	0.15
PubCons	0.87 (1.04)	1.19 (1.26)	0.90 (1.11)	0.31		0.0017		0.12	0.25		0.19	0.06

Note

* denotes significant differences between conditions after correcting for multiple comparisons (Bonferroni correction).

### Prediction/Classification of lucid dreams

In half 1 of the dataset, we first used binary logistic regression to predict lucidity from content dimensions of dreams. As shown in S1 Table, the variables with the highest predictive values (regression coefficients) were positive emotion, cognitive clarity, sensory vividness and control (all *p*<0.005). Next, in half 2 of the dataset, we evaluated whether we could classify dreams as lucid or non-lucid using Bayesian combination of these variables (see Methods: *Bayesian classification*). Control as a single indicator yielded 73% Bayesian classification accuracy of lucidity; combining control and positive emotion yielded 86.1% accuracy; combining control, positive emotion and cognitive clarity yielded 92.5% accuracy; combining control, positive emotion, cognitive clarity and sensory vividness yielded 95.5% accuracy (S2 Table).

### Adverse effects

Galantamine was generally well-tolerated with a total of 14 participants (12%) reporting mild side effects on an active dose: 12 (10%) and 11 (9%) with the 8 mg and 4 mg doses respectively (note that most participants who reported side effects reported them on both doses), compared to 4 (3%) with placebo. Side effects were more commonly reported for both the 4 mg dose (*p* = 0.039) and 8 mg dose (*p* = 0.057) compared to placebo, and did not significantly differ between doses (*p* = 1). Reports included mild gastrointestinal upset, insomnia and next-day fatigue (S2 Appendix).

## Discussion

Together our results show that galantamine substantially and significantly increases the frequency of lucid dreaming. The double blind and placebo-controlled design of this study provides strong evidence that cholinergic enhancement with galantamine causally increases the frequency of lucid dreams in a dose-related manner. Increased incidence of lucid dreaming was observed for both 4 mg (27%) and 8 mg (42%) doses compared to 14% for the active placebo, with an increased incidence of lucid dreams in the 8 mg dose compared to 4 mg dose. In addition to assessing galantamine’s influence on lucid dreams, our study tested an integrated protocol for inducing lucid dreams, which combined some of the most effective existing methods of lucid dream induction reported in previous studies (including sleep interruption [18, 32] and the Mnemonic Induction of Lucid Dreams (MILD) technique [7]) together with cholinergic enhancement. This combined protocol resulted in a total of 69 out of 121 participants (57%) successfully having a lucid dream on at least one out of two nights on an active dose of galantamine. This protocol is one of the most effective methods for inducing lucid dreams known to-date, and holds promise for making lucid dreaming available to a wider population.

The mechanism by which AChE inhibition results in lucid dreams is not understood at this time. Acetylcholinesterase inhibitors (AChEls) inhibit the normal metabolic inactivation of acetylcholine (ACh) by inhibiting the enzyme, acetylcholinesterase (AChE), leading to accumulation of ACh. It is known that ACh and its agonists as well as AChE and its inhibitors/antagonists are involved with REM sleep [22, 23, 33]. Specifically, evidence suggests that REM sleep is modulated by acetylcholine and controlled by cholinergic neurons in the brainstem [24, 34]. For example, microinjection of the ACh agonist carbachol in the pons elicits extended periods of REM sleep, and many of the neurons critical to REM sleep are responsive to ACh [24, 33]. Furthermore, galantamine in particular has been associated with shortened REM latency and increased phasic activity, including REM density [25]. Notably, AChEls are also commonly used to enhance memory in patients suffering from Alzheimer's disease, and have shown promise in managing the symptoms of mild cognitive impairment [35]. Galantamine belongs to a class of drugs that includes donepezil (Aricept®) and rivastigmine (Exelon®), which are frequently associated with the reported side effect of an increase in the vividness or intensity of dreams [36, 37].

In general, therefore, the increased prevalence of lucid dreams caused by galantamine is likely to be related to its effects on cholinergic receptor activity during REM sleep, which could include one or several different mechanisms, including the reduction in REM sleep latency, or more likely, increased phasic activity, which our previous research has shown to be strongly associated with lucid dreaming [21]. The improvements in memory associated with galantamine may also contribute to increased propensity for lucid dreams, for example by increasing one’s ability to remember to recognize one is dreaming, which is a prospective memory task. Cholinergic modulation has also been shown to bias attention and behavior toward top-down goal directed responses, and away from bottom-up, habitual, and otherwise pre-potent responses [38, 39]. In the context of dreaming, the bottom-up, habitual responses to dream content will likely be the notoriously nonlucid responses of ordinary dreaming. That means taking experienced content at face value, as if real, and engaged or ignored, as it appears relevant or not, to the goal-related activity one supposes one is pursuing within the given situation. However, experienced content need not be taken at face value, and instead can be recognized as *dream* content. If the dreamer is sleeping with the mental set of becoming lucid by recognizing dream content predictive of dreaming [4], cholinergic modulation should favor attaining this goal via the top-down facilitation effect.

However, as AChEls also influence systemic norepinephrine, dopamine and serotonin [40, 41], another possible explanation is that the impact of AChEI on lucidity could be entirely, or partially, indirect, and lucid dreaming could be causally linked to the aminergic modulation that occurs as a corollary to the cholinergic modulation brought on by AChEl [42]. For instance, there is evidence that galantamine in particular enhances dopaminergic neurotransmission [43] and dopamine has been shown to enhance metacognition and conscious self-monitoring [44], which could potentially increase lucid dreaming. Future research will be needed for a better understanding of how the neuromodulatory changes brought on by AChEIs precisely facilitate the emergence of the higher-order consciousness and cognitive skills associated with lucid dreaming.

In addition to its effect on lucidity, galantamine was associated with increased recall, sensory vividness, bizarreness and complexity of dreams. We expected that galantamine would have a positive effect on memory given that, as noted above, AChEIs are used to treat memory deficits associated with Alzheimer's disease and mild cognitive impairment. As discussed above, evidence suggests that REM sleep is modulated by acetylcholine and that galantamine results in increased phasic activity during REM sleep [25]. The increased sensory vividness, bizarreness and complexity of dreams on galantamine are therefore consistent with the general intensification of REM sleep (and associated dreaming) triggered by the cholinergic enhancement [25].

Lucid dreams overall were associated with significantly higher levels of recall, cognitive clarity, control, positive emotion, sensory vividness and self-reflection on one’s thoughts and feelings compared to non-lucid dreams. This finding replicates the largest previous study of lucid dream phenomenology [45], which analyzed self-rated content scales from 699 reports provided by 52 relatively experienced lucid dreamers and found that lucid dreams had significantly higher levels of control, positive emotions, visual vividness and clarity of thinking. Our findings are also broadly consistent with a recent study comparing lucid and non-lucid dream phenomenology by Voss et al. [46], which found that lucid dreams had greater amounts of control, self-reflective thought, memory, and positive emotion. Our results suggest that, aside from the state-consciousness that defines being lucid (the explicit awareness of being in a dream), lucid dreams are best characterized by increased control, cognitive clarity, positive emotion and sensory vividness. Bayesian combination of these four dimensions allowed us to classify lucid dreams with greater than 95% accuracy, indicating that dreams that are scored high on all of these features are very likely to be lucid. Nevertheless, we would like to emphasize that increased self-ratings on these (or other) dream features is not sufficient to establish whether an individual was lucid. Indeed, it is possible that some dreams could be high on all of these features but still be non-lucid, which is consistent with the fact that our classification was less than 100%. It is beyond the scope of the current analysis to undertake a more detailed investigation of differences in lucid and non-lucid dream phenomenology. However, there are currently very few systematic studies of content differences between lucid and non-lucid dreams, and future research on this question would be welcome.

Two recent studies have attempted to induce lucid dreams with electrical stimulation of the brain and it might be asked how these techniques compare with the current approach. In one study, Stumbrys et al. [12] applied either tDCS or sham stimulation (counterbalanced across nights) to the frontal cortex during REM sleep. 7 out of 109 dreams were classified as having a clear indication of lucidity by a judge: 3 from sham stimulation and 4 from tDCS stimulation. As the difference of only 1 lucid dream between stimulation and sham conditions is well within any reasonable estimate of noise, overall tDCS as tested in this study appears to be ineffective for inducing lucid dreams. In another study, Voss et al. [13] reported that by applying transcranial alternating current stimulation (tACS) in the gamma frequency range over the frontal cortex, lucid dreams could be induced with a success rate of 58% under 25 Hz and 77% under 40 Hz stimulation. However, trials were arbitrarily classified as lucid solely on “elevated ratings (>mean + 2 s.e.) on either or both of the LuCiD scale factors insight and dissociation”. Voss et al. neither reported content analyses of the dream reports nor did they verify lucid dreaming through volitional eye-movement signaling. Dissociation in the sense of adopting a 3^rd^ person perspective has never been considered a defining feature of lucid dreams in the literature on the topic [4, 5, 47–49], and it is therefore highly controversial to classify dreams as lucid based solely on higher ratings of dissociation. Moreover, Voss et al. did not report how many dreams met their criteria for dissociation only. While the insight subscale corresponds more closely to what other researchers as well as the public refer to as lucid dreaming, it is unfortunately not possible to determine unambiguously from scores on any of the questionnaire items whether an individual was lucid. Perhaps the largest issue with the inference that the stimulation resulted in lucid dreams was related to how the scale anchors ranged from 0 (strongly disagree) to 5 (strongly agree), yet mean response scores in the 25 and 40 Hz stimulation conditions ranged from ~0.5–1.0, meaning that on average participants reported that they *disagreed* that their dream content contained insight and dissociation. Overall, the study did not show that tACS induced lucid dreams and it is too early to tell how effective tACS will be for lucid dream induction. Despite these considerations, we believe that follow-up studies using various methods of brain stimulation could be a fruitful direction for future research if properly combined with training in the mental set for induction.

Individuals with high levels of dream recall, avid interest in lucid dreaming, and motivation to have lucid dreams are the kind of participants we expect to give similar results following the protocol for lucid dream induction evaluated here. It is again worth emphasizing that all of the statements regarding the effectiveness of galantamine in stimulating lucid dreams are in the specific context of a lucid dreaming training program in Hawaii, which teaches participants to cultivate effective mental sets for lucid dream induction. That is not to say that this protocol could not facilitate high rates of lucid dreaming in other motivated groups. Indeed, there are several factors that suggest that this protocol could be useful to a wide population. First, our study sample was comprised of individuals from diverse backgrounds who came from all over the world to attend the workshops, and included a wide age range (19–75 years). Our data suggest that galantamine is effective for inducing lucid dreams for adults of all ages and both sexes when combined with sleep interruption and MILD. Furthermore, while individuals who had a lucid dream on an active dose of galantamine reported higher baseline rates of lucid dreaming, the data show that previous experience with lucid dreaming is not necessary. Indeed, of the ten individuals who reported never experiencing a lucid dream before the study, 40% were able to have their first lucid dream ever in a single nap with galantamine (8 mg), a success rate that was not different from the rest of the sample.

As noted, a primary aim of this study was to develop a protocol for inducing lucid dreams by combining the most effective methods known for lucid dream induction together with cholinergic enhancement. However, because these methods were not tested here in isolation from each other, the precise quantitative effect of MILD and sleep interruption in this study remains unclear. Future studies might consider comparing, for example, MILD alone to MILD plus sleep interruption to MILD plus galantamine to MILD plus sleep interruption plus galantamine in order to address this question. Likewise there were several additional aspects of the training program likely to contribute in some degree to increased lucid dream frequency, from group discussion of lucid dreams to being in a unique environment, which together might be termed “set and setting”. The combined effect of these factors can be estimated by comparing lucid dream frequency on the placebo dose (14%) to the 4% estimated baseline rate. However, any influence of these factors does not impact the dose-related effect of galantamine on lucidity established by comparison to the placebo control condition.

Because the experiment was not conducted in a sleep laboratory, we therefore do not have physiological recordings of lucid dreams or polysomnographic validation of participants’ sleep stage. However, several aspects of our methodology lend weight to the authenticity of participants’ reports of lucid dreams. First, participants developed a thorough conceptual understanding of the meaning of lucid dreaming through lectures and discussion sections in the pre-experiment workshop, limiting any errors in self-reports due to misunderstandings of what constitutes a lucid dream. Second, as discussed above, participants explicitly reported whether they had a lucid dream (and if so what type), in contrast to other studies that have inferred lucidity based on measures of dream content (i.e., [13]). Finally, full written and oral reports of lucid dreams were collected, which had to include a clear indication of lucidity including explicit mention of knowing the current state was a dream. Together these factors provide confidence in the reliability of participants’ reports of lucid dreams in this study.

Due to the fact that sleep cycles could not be objectively assessed with a sleep hypnogram, the timing of the sleep interruption period had to be approximated rather than timed to specific REM cycles. Future research might test whether the impact of galantamine and sleep interruption can be further optimized by comparing protocols administered after specific sleep cycles, at specific placement with respect to sleep cycles, as well as the optimal activity to engage in during the sleep interruption period. The adverse effects reported in the study were difficulty falling back to sleep (N = 5) and mild gastrointestinal symptoms, including nausea (N = 5). While these side effects were mild and their incidence low in our sample, future studies might further reduce the incidence of adverse reactions by testing and screening for cholinergic sensitivity or use of anti-nausea medication. Future studies might also test whether a higher dose of galantamine (e.g., 10 or 12 mg) would further increase lucid dream frequency without increasing side effects.

Altogether the current results show that galantamine substantially increases the frequency of lucid dreams with a minimal side effect profile, suggesting it is well-placed as a method for inducing lucid dreams, and has the potential to make lucid dreaming available to a wider population. While more studies documenting the safety profile of this substance are needed, the potential of making lucid dreaming widely accessible also has implications for scientific research. As one of the challenges for conducting scientific research on lucid dreaming is its relative infrequency, making the state more accessible would increase the efficiency of the collection of larger datasets, which would greatly facilitate research in this field.

## Supporting information

S1 Appendix Examples of narrative reports classified as lucid dreams. (DOCX)

S2 Appendix Supplementary procedures and results. (DOCX)

S1 Table Prediction of lucidity from dimensions of consciousness (DIM) using binary logistic regression rank-ordered by predictive value. (DOCX)

S2 Table Bayesian classification of lucidity from DIMs. (DOCX)
