# Supplementary material for: Pre-sleep treatment with galantamine stimulates lucid dreaming: A double-blind, placebo-controlled, crossover study
Source: PLoS One. 2018 Aug 8;13(8):e0201246. doi: 10.1371/journal.pone.0201246 (PMC6082533; doi:10.1371/journal.pone.0201246)
Supplement: S1 Appendix — (DOCX) [file pone.0201246.s001.docx]

**Supplementary Appendix 1: Examples of narrative reports classified as lucid dreams**

**Examples of reports classified as dream-initiated lucid dreams (DILDs)**

I’m dreaming I’m in CVS with friends at home. It’s larger than usual and the atmosphere is somewhat fun. As I passed some rollerblades on the shelf, I remember my waking life wish to roller skate or skateboard in a megastore. I thought to myself, if I were lucid I could go rollerblading in here! *Then I realized I was dreaming.* My friends and I began rollerblading down the aisles. I was having so much fun that I lost lucidity and became reabsorbed in the dream plot. My dream characters convinced me it was time to go. I felt guilty that I stole the rollerblades from CVS and vowed to return them the next day.

25-year-old female, March 2010, Night 3 [4 mg dose]

I was at J.’s house in New Zealand and he was showing me all his animals and I kept thinking I have to remember something. Then he showed me his donkeys. While we were looking at them they all started climbing a telephone pole and I got nervous they were going to fall on me. And I thought, “*Wait a minute, this is a dream!* I can just fly higher than they are and they won’t fall on me”.

22-year-old female, October 2012, Night 1 [4 mg dose]

Driving in a car and could not see the road. It was raining. The windshield was getting foggy. Headlights were hitting me. I remember thinking I am going to have an accident. Then it hit me that I’m dreaming and I shout, *“This is a dream!”* I’m at first afraid to let go of the steering wheel but do it anyway it feels great. The car melts away and I seem to keep going without it into blackness.

58-year-old male, October 2012, Night 3 [4 mg dose]

I’m stressed about being at work. I am scolded by my boss for leaving my on-duty post. Numerous dreamsigns appeared: an incorrect vehicle, no bells sounding to announce the start of classes, being naked, and not being able to find my classroom. I question whether or not I might be dreaming and “reality check” by looking at my digital watch a few times. *Now lucid, I tell my friend dream character “I’m dreaming”.* My friend is thinking this has to be impossible, so I explain lucid dreaming to her and how she can do it. She then runs off and begins mistreating dream characters. I explained that this is not conducive to lucid dreaming, and tell her about being kind to the dream characters. I have a false awakening, where I tell David and Don about my dream. We laughed about the dreamsigns.

31-year-old male, May 2015, Night 2 [0 mg dose]

**Examples of reports classified as wake-initiated lucid dreams (WILDs)**

*I went straight from sleep into lucidity*. I felt someone really close to me and it was very comforting. They were massaging me, especially my head. I said, “oh that is nice.” I smelled an orange smell quite strongly. I realized that I had the intention to fly so I did, which as always was elating. The person next to me became more intimate and we had a warm sexual encounter after which I awoke.

64-year-old female, March 2010, Night 3 [8 mg dose]

I woke myself at 3:30 AM four about 50 minutes. I was awake–10 minutes of reading, later listened to relaxation, did visualizing using mild, and combining the hypnagogic stage and visualization. I got some hypnagogic images that later turned into a dream. *I continue to be lucid this whole time*. *It was very clear that it is the dream* (As I was aware all the time, could fly etc., with full control of this “crazy environment”). I continue to be lucid for about two hours. When the dream started to fade I spun myself around to attempt to continue with the lucid dream [*prolonging technique*]. During the dream I visited many places, and did many “experiments” (reading, tasting, drawing shapes, etc.).

45-year-old male, September 2012, Night 1 [8 mg dose]
