# Supplementary material for: Pre-sleep treatment with galantamine stimulates lucid dreaming: A double-blind, placebo-controlled, crossover study
Source: PLoS One. 2018 Aug 8;13(8):e0201246. doi: 10.1371/journal.pone.0201246 (PMC6082533; doi:10.1371/journal.pone.0201246)
Supplement: S2 Appendix — (DOCX) [file pone.0201246.s002.docx]

**Supplementary Appendix 2: Supplementary Procedures and Results**

*Galantamine supplement*

We sourced Galantamine hydrobromide from Life Enhancement Products (Galantamind™) due to its consistent availability over the several years of our study. Galantamind also contains small amounts of choline and vitamin B5, but we did not expect those ingredients to affect our results. Nevertheless, we performed an experiment to test whether there was any difference in efficacy between the Galantamind formulation, and a supplement from another source containing only an identical amount of galantamine. As predicted, there was no difference in the frequency of lucid dreams following an active dose of galantamine (*N*=14, *M*=71%) compared to Galantamind® (*N*=13, *M*=69%) [*t*(25)=0.12, *p*=0.91].

*Sleep masks*

94 self-selected participants also wore a sleep mask which recorded physiological variables and provided additional memory cues during sleep [[11](#_ENREF_11), [13](#_ENREF_13)]. Analysis of the physiological variables will be reported in a separate publication. Participants who opted to sleep with the mask were required to use it on all three nights of the experiment after the sleep interruption period, ensuring any effects of the sleep masks would be independent of the within-subjects, cross-over effects of the galantamine intervention. The device consists of a soft, comfortable sleep mask worn over the eyes, which contains LED lights and an eye movement detection apparatus housed inside the mask. As noted above, the device detects rapid eye movements and then emits flashes of light over the sleeper’s eyes during REM sleep, which are often incorporated into the ongoing dream, thereby providing memory cues to prompt lucidity [[13](#_ENREF_13)].

Overall, participants reported a cue from the sleep mask in the content of 29 dreams during the experiment: 12 during nonlucid dreams and 17 during lucid dreams. Cues were more commonly reported in lucid compared to nonlucid dreams (*ß*=0.16, *p*=0.001), and were more frequently reported on the 8 mg dose (*ß*=0.09, *p*=0.034) but not on the 4 mg dose (*ß*=0.02, *p*=0.55) compared to placebo. Participants who used the sleep masks were more likely to have a lucid dream overall during the experiment (*t*(119)=2.15, *p*=0.03) and marginally more likely to have a lucid dream on an active dose of galantamine (*t*(119)=1.95, *p*=0.053). However, due to the self-selection of participants it is not clear whether this effect was due to pre-existing differences between groups or an independent effect of the sleep masks.

*Sleep interruption period*

There were 42 nights in which participants reported a sleep interruption period less than the recommended 30 minutes: 15 on placebo, 13 on the 4 mg dose and 15 on the 8 mg dose. Participants were more likely to have a lucid dream if they engaged in at least 30 minutes of sleep interruption (*ß* =0.11, *p*=0.03, one-tailed). There was no relationship between the likelihood of having a lucid dream and the total time between the end of the sleep interruption period and rise time (non-lucid: *M*=201.41, *SD*=65.99; lucid: *M*=195.90, *SD*=63.68) [*ß* = -0.0002, *p*=0.55].

*Somatic sensations*

Odd somatic sensations (e.g., sleep paralysis, tingling, vibrations) were more commonly reported during lucid compared to non-lucid dreams (*ß* =0.92, *p*<0.0001) for both DILDs (*ß* =0.83, *p*<0.0001) and WILDs (*ß* =1.17, *p*<0.0001). Odd somatic sensations were more common following WILDs compared to DILDs (*ß* =0.34, *p*=0.05, one-tailed). No differences in odd somatic sensation were observed between placebo and either the 4 mg (*ß* = -0.01 *p*=0.88) or 8 mg galantamine dose (*ß* =0.09, *p*=0.37).

*Adverse effects*

Five participants (4%) reported nausea on an active dose: three (3%) on the 8 mg dose (causing attrition in one participant), and two (2%) on the 4 mg dose, one (1%) of whom also reported it on placebo. Insomnia was reported by seven participants (6%): four (3%) on the 8 mg dose, five (4%) on the 4 mg dose, and one participant (1%) on the 0 mg dose. Three participants (2%) were unable to return to sleep following the sleep interruption period, two (2%) who took the 8 mg dose, and one (1%) who took the 4 mg dose. Fatigue was reported by three participants (2%): three (3%) on 8 mg, one (1%) on 4 mg, and one (1%) on 0 mg of galantamine.
