# Supplementary material for: Pre-sleep treatment with galantamine stimulates lucid dreaming: A double-blind, placebo-controlled, crossover study
Source: PLoS One. 2018 Aug 8;13(8):e0201246. doi: 10.1371/journal.pone.0201246 (PMC6082533; doi:10.1371/journal.pone.0201246)
Supplement: S1 Table — (DOCX) [file pone.0201246.s003.docx]

**Table S1. Prediction of lucidity from dimensions of consciousness**

**(DIM) using binary logistic regression rank-ordered by predictive value.**

| **DIM** | ***Beta*** | **Std. Error** | **z value** | **p-value** |
| --- | --- | --- | --- | --- |
| Pos Emotion | 1.18 | 0.23 | 4.96 | 7 x 10^-7^ |
| Clarity | 1.06 | 0.22 | 4.67 | 4 x 10^-6^ |
| Vividness | 0.66 | 0.21 | 3.06 | 0.002 |
| Control | 2.27 | 0.78 | 2.88 | 0.003 |
| Complexity | 0.39 | 0.16 | 2.38 | 0.01 |
| Self-reflection | 0.39 | 0.19 | 2.07 | 0.03 |
| Bizarreness | 0.24 | 0.14 | 1.66 | 0.09 |
| PubCons | 0.05 | 0.15 | 0.35 | 0.72 |
| Neg Emotion | -0.15 | 0.16 | -0.96 | 0.33 |
| Recall | 1.00 | 0.26 | 3.75 | 0.001 |
