# Supplementary material for: Pre-sleep treatment with galantamine stimulates lucid dreaming: A double-blind, placebo-controlled, crossover study
Source: PLoS One. 2018 Aug 8;13(8):e0201246. doi: 10.1371/journal.pone.0201246 (PMC6082533; doi:10.1371/journal.pone.0201246)
Supplement: S2 Table — (DOCX) [file pone.0201246.s004.docx]

**Table S2. Bayesian classification of lucidity from DIMs.**

| **Indicators** | **Sensitivity** | **Specificity** | **BPP** |  |  |
| --- | --- | --- | --- | --- | --- |
| Positive Emotion | 0.54 | 0.76 | 0.41 |  |  |
| Clarity | 0.61 | 0.70 | 0.38 |  |  |
| Vividness | 0.77 | 0.55 | 0.34 |  |  |
| Control | 0.39 | 0.96 | 0.73 |  |  |
| Complexity | 0.53 | 0.63 | 0.30 |  |  |
| Self-reflection | 0.30 | 0.87 | 0.41 |  |  |
| Bizarreness | 0.46 | 0.76 | 0.36 |  |  |
| Public Consciousness | 0.19 | 0.90 | 0.38 |  |  |
| Neg Emotion (low) | 0.75 | 0.11 | 0.20 |  |  |
| Recall | 0.74 | 0.58 | 0.35 |  |  |
|  |  |  |  |  |  |
| **Bayesian Classification**  **(Combined Indicators)** |  |  |  |  | **BPP** |
| Control |  |  |  |  | 0.73 |
| Control + Positive Emotion |  |  |  |  | 0.860 |
| Control + Positive Emotion + Clarity | | |  |  | 0.925 |
| Control + Positive Emotion + Vividness | | |  |  | 0.913 |
| Control + Positive Emotion + Complexity | | | |  | 0.898 |
| Control + Positive Emotion + Self-reflection | | | |  | 0.934 |
| Control + Positive Emotion + Clarity + Vividness | | | |  | 0.955 |
| Combination of all indicators | |  |  |  | 0.997 |
